# Resection of an intra-operative ruptured hepatocellular carcinoma with continuous pringle maneuver and in situ hypothermic perfusion through the inferior mesenteric vein - a case report

**DOI:** 10.1186/1477-7819-11-2

**Published:** 2013-01-09

**Authors:** Yueh-Ming Lin, Li-Wei Chiang, Shih-Ho Wang, Chih-Che Lin, Chao-Long Chen, Carlos A Millan, Chih-Chi Wang

**Affiliations:** 1 Division of General Surgery, Department of Surgery, Kaohsiung Chang Gung Memorial Hospital and Chang Gung University College of Medicine, 123 Ta-Pei Road, Niao-Sung, Kaohsiung, Taiwan

**Keywords:** Hepatectomy, In situ hypothermic perfusion, Continuous pringle maneuver

## Abstract

Intra-operative tumor rupture is a serious complication during resection of large hepatocellular carcinoma (HCC) leading to more blood loss. We report our experience in applying continuous Pringle maneuver with in situ hypothermic perfusion via inferior mesenteric vein catheterization to the portal vein of the remnant liver for resection during an extended left lobectomy of a large HCC which ruptured intraoperatively. Using this method, we successfully managed the patient without any further morbidity. This technique provides easier accessibility of in situ perfusion, decreases operative blood loss and prevents warm ischemic injury to the remnant liver during parenchymal transection. This method could be effective for the resection of large ruptured HCC.

## Background

Hepatic resection remains the optimal choice that offers long-term survival for patients with hepatocellular carcinoma (HCC). Various types of hepatic resection have been created and modified over the years to achieve curative resection with adequate margins [1]. Remnant liver function and operative blood loss are two major concerns during hepatic resection. It can be calculated pre-operatively with detailed imaging evaluation. There are various methods for hepatic vascular control, namely, Pringle maneuver (PM), *in situ* hypothermic perfusion (ISHP), and total hepatic vascular exclusion (HVE) [2]. Intra-operative iatrogenic rupture of HCC, which can occur during hepatic resection, leads to adverse effects such as increased intra-operative blood loss [3].

Hypothermic liver perfusion has been shown to attenuate hepatic ischemic injury in large animals [4,5] and humans [6,7]. The ISHP technique appears to be a useful adjunct to radical hepatobiliary tumor excision, which requires total HVE and major vascular reconstruction [8]. Recent studies have shown that continuous inflow occlusion coupled with *in situ* liver cooling can be used safely, even among patients with chronic liver disease [9,10]. Therefore, continuous inflow control plus ISHP is indicated for large HCC, which can be associated with more intra-operative blood loss and a longer operating time.

Herein, we report a case of an intra-operative rupture of a large HCC that was resected using continuous PM with ISHP via inferior mesenteric vein (IMV) catheterization. We believe this is the first case report in English literature about ISHP via IMV catheterization to the portal vein (PV) of the remnant side.

## Case presentation

**Figure 1:**
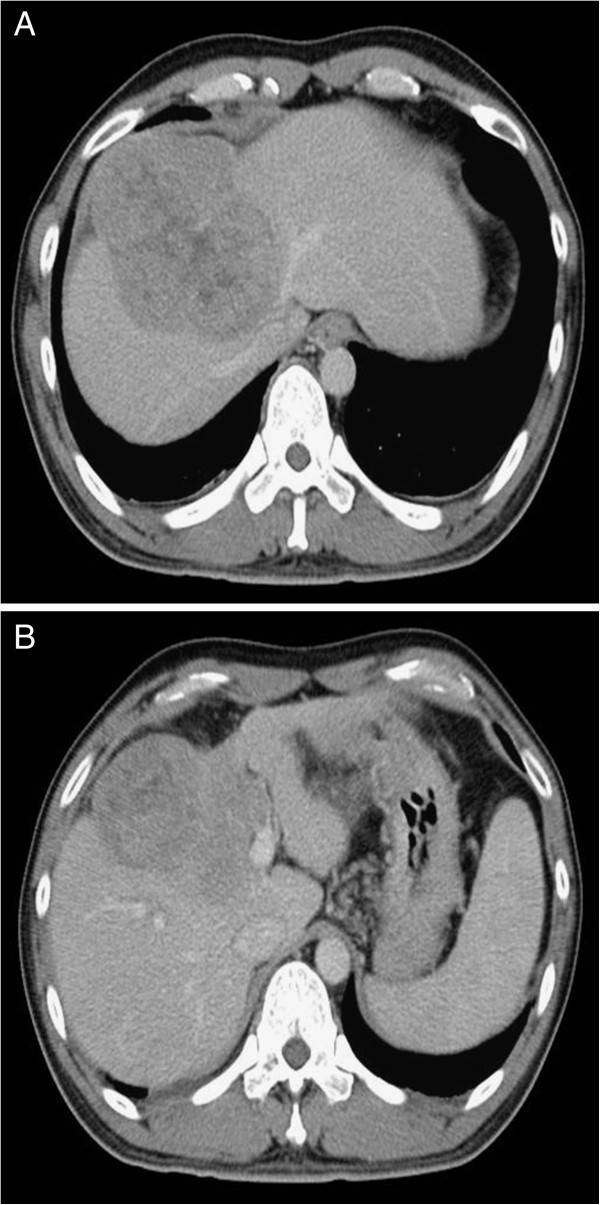
**Computed tomography (CT) scan images before surgery.** (**A**) Large liver tumor with diaphragmatic invasion and occlusion of the middle hepatic vein. (**B**) Large liver tumor with tumor thrombus in the left portal vein.

A 38-year-old man with history of chronic hepatitis B was referred to our hospital for liver tumor management. The triphasic helical computed tomography (CT) scan revealed an 11-cm mass in segment 4 and 8 with early enhancement and early washout and a thrombus was detected in the left PV (Figure 1). Angiography disclosed the main tumor and two additional tumors in the right lobe. Preoperative laboratory data showed an indocyanine green (ICG) test of 7.2%, aspartate aminotransferase (AST) 63 U/L, alanine aminotransferase (ALT) 48 U/L, total bilirubin level 0.9 mg/dL, and alpha-fetoprotein (AFP) 303,145 ng/ml. The operative plan was to perform an extended left lobectomy for the main tumor, followed by transcatheter arterial embolization (TAE) for the right-sided liver tumors.

Due to severe adhesion of the tumor to the surrounding tissues, tumor rupture occurred during dissection. Compression of the tumor and PM was applied immediately to stop the bleeding. A long warm ischemic time was expected for complex hepatectomy under PM. The main PV was dissected and looped with a Rumel tourniquet; following this, a cannula (8 French) was inserted into the right PV through a small venotomy in the IMV, and was secured by a silk tie. Subsequently, the hilum was continuously clamped except for the right PV infusion of cold 4°C lactated Ringer’s solution, which was instituted at an initial rate of 500 mL/h. The infusion rate was then adjusted to keep the liver temperature between 26°C and 30°C, and the body temperature between 35°C and 37°C. Parenchymal transection was carried out by using a combination of techniques, including Kelly clamp fracturing and a Cavitron Ultrasonic Surgical Aspirator (CUSA system 200; Valleylab Inc., Boulder, CO, USA).

**Figure 2:**
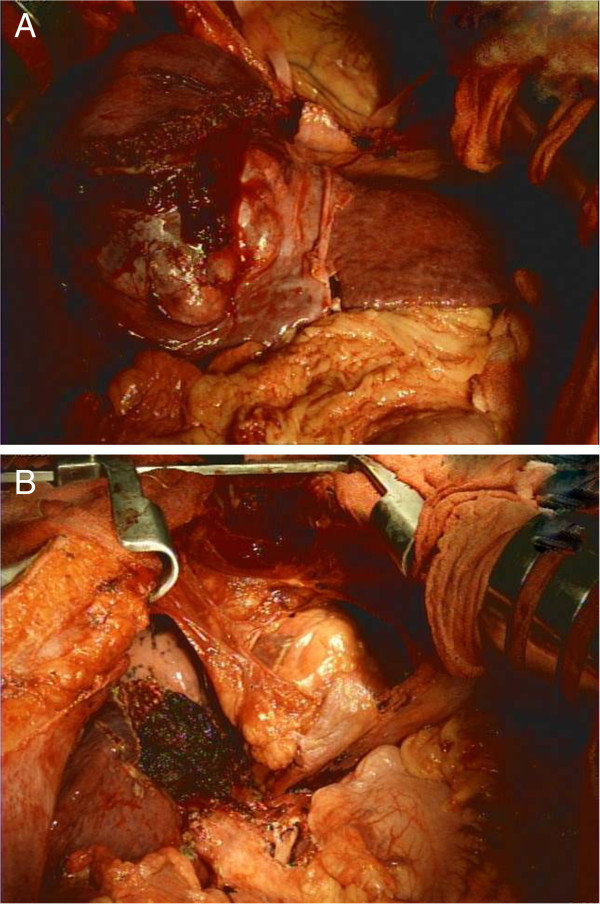
**Intra-operative images.** (**A**) Large liver tumor with adhesion to the diaphragm and pericardium. (**B**) The transaction surface after removal of the large liver tumor.

Parenchymal transection was aimed at achieving a 5 mm resection margin. The average core liver temperature during ISHP measured by a needle probe (model YFi-100A, Yu-Fong, Taiwan) placed at a depth of 2 to 3 cm into the liver parenchyma of the remnant side was 27.9°C (range 26.0°C to 30.4°C). The mean body temperature was 36.0°C (range 35.6°C to 36.3°C) during ISHP. The ISHP time was 90 minutes and the parenchymal transection time was 63 minutes. Blood loss was 800 mL. The total operation time was 11 hours 34 minutes. The representative operative images are shown in Figure 2.

**Figure 3:**
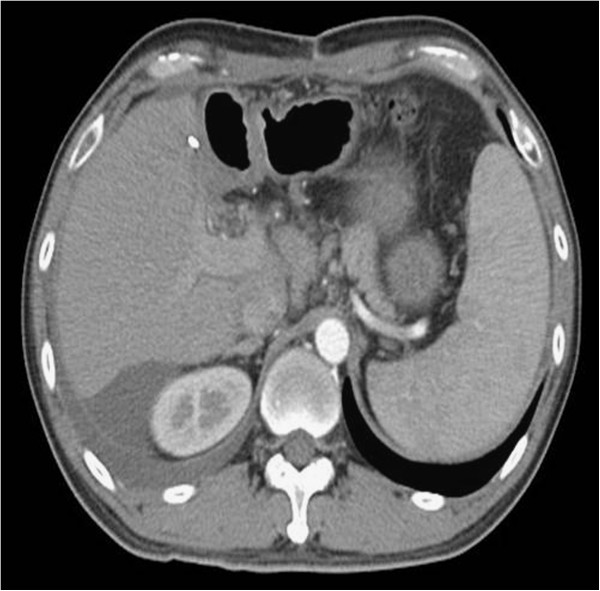
Computed tomography (CT) scan image after surgery.

The postoperative laboratory data were AST 250 U/L, ALT 267 U/L, and total bilirubin 3.1 mg/dL on postoperative day (POD) 1; AST 145 U/L, ALT 164 U/L, and total bilirubin 2.5 mg/dL on POD 2, and AST 84 U/L, ALT 94 U/L, and total bilirubin 3.9 mg/dL on POD 3. Liver function tests returned to baseline level on POD 5: AST 53 U/L, ALT 47 U/L, and total bilrubin 1.3 mg/dL. Peri-operative serum creatinine levels were within normal limits. The follow-up postoperative AFP level was 1488 ng/mL on POD 72. The postoperative clinical course was uneventful and there were no major complications. Figure 3 shows the postoperative image. Residual tumors at the right side of the liver were managed by TAE after complete recovery following surgery. Recurrent HCCs in the remnant liver were detected 130 days after surgery and were treated by additional TAE.

## Discussion and conclusions

In this report, an intra-operative ruptured large HCC was successfully managed by continuous PM with ISHP via IMV catheterization. The technique of ISHP of the liver was described by Fortner *et al*. in 1974 [11]. Hypothermia diminishes the metabolic rate, slows the cellular degradation process of intracellular organs, and decreases the lesions of membranes [12]. One study suggests that when hypothermic perfusion is used in addition to TVE, it provides significantly better tolerance to ischemia, better liver function, and more importantly, a significantly lower number of complications [12].

However, there is still debate about whether there is a significant advantage of continuous PM coupled with ISHP, over intermittent PM [13]. Some reports have demonstrated that continuous inflow occlusion coupled with *in situ* liver cooling is a safe procedure with the benefit of being able to prolong the ischemic time, even for patients with chronic liver disease [9,10,13]. Lowering liver temperature between 26°C and 30°C allows the procedure to go on for up to 140 minutes [13]. Torzilli *et al*. [14] reported the safety of systematic prolonged intermittent PM for hepatectomies exceeding 120 minutes. This report seems to be encouraging for extensive liver resection; however, there is still concern about poor tolerance of the ischemic insult to the liver with underlying disease. Hence, the resection method of large HCC, which carries the possibility of major blood loss and even tumor rupture, should be planned in detail before surgery.

When the *in situ* liver cooling method is used, cannulation of the PV of the remnant side is usually performed through a small venotomy near the bifurcation of the PV. It is not uncommon for the catheter to be dislodged during liver mobilization for parenchymal transection. It is difficult to perform venotomy for catheterization of the PV under the Satinsky clamp during PM.

The IMV in the region of the bifurcation of the aorta deviates to the left and upward as it passes beneath the pancreas to join the splenic vein, and is easily identified during laparotomy. According to our experience, the major advantages of using the IMV for catheter placement are easier accessibility and lower risk of catheter dislodgement compared to management by means of PV cannulation.

In conclusion, when dealing with large HCC, which may carry the risk of more blood loss and longer transection time, continuous PM with ISHP via IMV catheterization to the remnant side may provide a suitable alternative method for parenchymal transection.

## Consent

Written informed consent was obtained from the patient for publication of this case report. A copy of the written consent is available for review by the Editor-in-Chief of this journal.

## Abbreviations

ALT: Alanine aminotransferase; AFP: Alpha-fetoprotein; AST: Aspartate aminotransferase; CT: Computed tomography; HCC: Hepatocellular carcinoma; ICG: Indocyanine green; PM: Pringle maneuver; ISHP: *In situ* hypothermic perfusion; HVE: Hepatic vascular exclusion; IMV: Inferior mesenteric vein; POD: Postoperative day; PV: Portal vein; TAE: Transcatheterarterial embolization; TVE: Total vascular exclusion.

## Competing interests

The authors declare that they have no competing interests.

## Authors’ contributions

YML, LWC: prepared and edited this manuscript, SHW, CCL, CAM: collection, analysis and interpretation of data CLC and CCW: study design, CCW: final approval of the version to be published. All authors read and approved the final manuscript.
